# Fatigue in myasthenia gravis: is it more than muscular weakness?

**DOI:** 10.1186/1471-2377-13-132

**Published:** 2013-10-03

**Authors:** Ahmed Elsais, Vegard B Wyller, Jon Håvard Loge, Emilia Kerty

**Affiliations:** 1 Department of Neurology, Oslo University Hospital, Rikshospitalet, Postbox 4950, Nydalen, 0424 Oslo, Norway; 2 Faculty of Medicine, University of Oslo, Oslo, Norway; 3 Department of Paediatrics, Oslo University Hospital, Oslo, Norway; 4 National Resource Centre for Late Effects after Cancer Treatment, Oslo University Hospital, Oslo, Norway

**Keywords:** Myasthenia gravis, Fatigue, Autonomic nervous system, Self report, Questionnaire, Acetylcholine esterase inhibitors

## Abstract

**Background:**

Few studies have focused on fatigue in myasthenia gravis (MG), and fatigue in relation to the autonomic system has never been systematically explored in these patients. The study aimed to document the prevalence of MG-related fatigue in ethnic Norwegians and to examine whether MG severity is associated with symptoms of autonomic disturbance, which in turn is associated with fatigue and functional disability.

**Methods:**

Eighty two of the 97 who fulfilled the study inclusion criteria participated in the study. Controls were 410 age- and sex-matched subjects drawn from a normative sample (n = 2136) representative of the Norwegian population. Bivariate analyses and multivariate linear regression analyses were used to assess associations between questionnaire-reported MG severity, symptoms of autonomic disturbance, fatigue (mental and physical) and functional disability.

**Results:**

Forty-four per cent (36/82) of patients fulfilled the criteria for fatigue compared with 22% (90/410) of controls (odds ratio 2.0; p = 0.003). Twenty-one per cent of patients (17/82) met the criteria for chronic fatigue *versus* 12% (48/410) of controls (odds ratio 1.96; p = 0.03). MG patients had higher total fatigue scores than controls (p < 0.001) and a high prevalence of autonomic symptoms, especially poor thermoregulation and sleep disturbance. According to multivariate analyses controlled for MG score, symptoms of autonomic disturbances were independently positively associated with fatigue (p < 0.001), and fatigue was independently negatively associated with functional level (p < 0.001).

**Conclusion:**

Norwegian ethnic patients with MG have higher levels of fatigue and a higher prevalence of chronic fatigue than controls, even in patients in full remission. MG severity is highly suggestive to be associated with symptoms of autonomic disturbance, which in turn is associated with fatigue and the level of functional disability.

## Background

Myasthenia gravis (MG) is a chronic autoimmune disease that affects the neuromuscular junction, causing reduced muscular strength and reduced endurance of repetitive muscle use. Fatigue has been reported in both neurological and non-neurological diseases, including MG, independent of muscle weakness [1,2]. The perception of fatigue is subjective and there is no consensus on an exact definition [3]. Because fatigue is a complex phenomenon and includes both physiological and psychological factors, a distinction has recently been made between fatigue as a subjective feeling of tiredness, lack of energy, and difficulty concentrating, and muscle fatigability defined as the difficulty initiating or sustaining muscle activities [4]. Chronic fatigue is commonly defined as fatigue above a certain level lasting for 6 months or more [5]. Persistent fatigue of this type has been studied extensively in the Chronic Fatigue Syndrome (CFS) and in some other chronic diseases. In this study, the characteristics of fatigue in CFS will be applied as a working definition of chronic fatigue in MG patients.

Muscle fatigability has been mentioned in most MG studies but only a few studies have investigated subjective fatigue in MG patients [6-8]. These studies have shown that MG patients have more fatigue than healthy controls [7,8]; in one study 82% of MG patients complained of fatigue and half of these also reported mental fatigue which could only partly be explained by muscle fatigability [7,8]. Another study found no direct association between fatigue and neurophysiologically proven muscle fatigability by use of decrement tests [8].

Using orthostatic stress and isometric muscle contraction as challenges, previous studies of CFS found that feelings of fatigue can be related to autonomic dysregulation [9,10] Autonomic dysfunction as a result of deficient regulatory mechanisms of the sympathetic nervous system in MG patients has been reported [11-13]. Kimura and coworkers suggested that cholinergic autonomic nerves may be affected in MG patients even in the absence of clinical signs of autonomic dysregulation [14]. Muscle and neuronal acetylcholine receptors (AChR) are structurally similar, and cross-immunity has been shown between the two types of AChR antibodies in MG patients who have the concomitant syndrome “autoimmune autonomic neuropathy”. This syndrome responded to acetylcholine esterase inhibitors (AChEI) [15], which could indicate that presynaptic impairment is an underlying cause of the autonomic disturbances in MG patients. However, fatigue has never been studied in relation to autonomic disturbances in MG patients.

We noted that MG patients often indicated a general feeling of lack of energy and trouble concentrating, but repeated clinical examinations showed no signs of muscular weakness in many of these patients. These fatigue symptoms did not improve after rest or the intake of AChEI. This observation is the background for the present study. Our primary goal was to test the hypothesis that MG severity is associated with symptoms of autonomic disturbance, and a secondary goal was to document the prevalence of fatigue in myasthenia gravis patients.

## Methods

### Study sample

The patient registry of Oslo University Hospital, Rikshospitalet, was used to identify ethnic Norwegian patients, aged 19–74 years who were diagnosed with acquired autoimmune MG between 1996 and 2006 (n = 125). Diagnosis was based on conventional diagnostic criteria; all patients had typical exertional muscle weakness, and at least two supportive laboratory results, such as a positive edrophonium test, and/or the presence of AChR antibodies in serum, and/or neurophysiological findings consistent with MG (decrement >10% at 3 Hz repetitive motor nerve stimulation and/or increased jitter on single-fibre electromyogram).

A detailed clinical neurological examination was performed at least twice during the year before participation by two of the authors (AE, EK), including a muscle power test of eye muscles (ptosis test and eye motility), bulbar muscles (swallow and speech), muscle power in extremities, neck and respiratory muscles.

Clinical grading according to the Myasthenia Gravis Foundation of America (MGFA) scale was performed on the basis of the patient’s symptomatology and clinical examination. Only patients with MGFA grade II or better during the year prior to the start of the study, regardless of the severity of their MG in the past, were invited to participate (n = 97). The inclusion criteria for the present study were a definitive MG diagnosis, clinical condition not worse than MGFA grade II during the last year before participation, and age between 19 and 74 years.

The statistical program SPSS 17.0 software (SPSS Inc., Chicago, IL, USA) was used to randomly draw five age- and sex-matched controls per eligible patient from normative data, representative of the Norwegian population (n = 2136, age 19–74) [16]. Information about age, body mass index (BMI), disease duration, comorbidities and use of medication (especially AChEI) was obtained from patient records. A questionnaire was sent to patients and controls regarding sociodemographic status, fatigue and duration of symptoms. MG patients also completed two self-reported questionnaires on severity of MG and symptoms of autonomic disturbance.

All study subjects signed informed consent forms. The study was approved by the appropriate independent hospital ethics committee.

### Questionnaires

Study subjects completed the Norwegian version of the FQ (Fatigue Questionnaire) [16,17], which assessed fatigue symptoms experienced during the past month compared with the last time the subject felt well. Seven items related to physical fatigue and four to mental fatigue. The FQ also included a question about symptom duration. Each of the 11 items had four response alternatives; “less than usual” = 0; “same as usual” = 1; “more than usual” = 2; and “much more than usual” = 3, with a maximum possible score of 33. The sum of responses to all items was designated the “Total fatigue level” with a possible range of scores between 0 and 33. The sum of responses to the seven physical items indicated the level of “physical fatigue” (score range 0–21). The sum of responses to the four mental items indicated the level of “mental fatigue” (score range 0–12).

Responses with alternatives were dichotomised; 0 and 1 = 0, 2 and 3 = 1. Substantial fatigue or “fatigue caseness” was defined as a sum score of dichotomised responses ≥ 4 and chronic fatigue was defined as a sum score of dichotomised responses ≥ 4 and symptom duration of ≥ 6 months [16].

The Myasthenia Gravis Questionnaire (MGQ) is a 25-item, disease-specific, patient-reported questionnaire [18,19]. The authors used the validated Swedish version [19], translating only a few terms into Norwegian (which is very similar to Swedish). The global MGQ score was the sum of scores for all items and ranged from 0 (maximal impairment) to 50 (absence of any impairment). The score of MGQ was validated as an outcome measure and was found to correlate highly with clinical status and MG severity [18,19].

The Autonomic Symptom Questionnaire is a validated instrument for assessing symptoms of autonomic dysfunction [20]. The Autonomic Symptom profile (ASP) used in the present study is a translation of an abridged version (22 selected items) of the validated questionnaire, and had been proven feasible in a previous study at our institution [21]. The 22 items assessed orthostatic intolerance, vasomotor function, sudomotor/thermoregulatory function, gastroenterologic function, pupillary function and sleep disturbance. The response alternatives were partly dichotomous (“yes” and “no”), and were partly scored from 1 to 5 on a Likert scale. In this study, the responses to all ASP items were dichotomised and assigned a value of 0 (symptom not/rarely present) or 1 (symptom often/always present). Autonomic symptom scores were the sum across all ASP items and were in the range 0–22. The ASP questionnaire also included three items that addressed social and physical disability on 1–5 Likert scales. The total disability score was the sum of these three items in the range 3–15. There was an Item assessing the symptoms of chronic fatigue syndrome (CFS) [22], an item assessing the level of physical activity during leisure time (total score range from 2–6), and an item to cover comorbidity with possible autonomic consequences either caused by the disease itself, hyper-tension, heart disease, diabetes, thyroid abnormalities, polyneuropathy, rheumatoid arthritis or their treatment. Comorbidity was expressed as none = 0, one disease = 1, and 2 or more = 2.

### Statistical analysis

**Figure 1:**
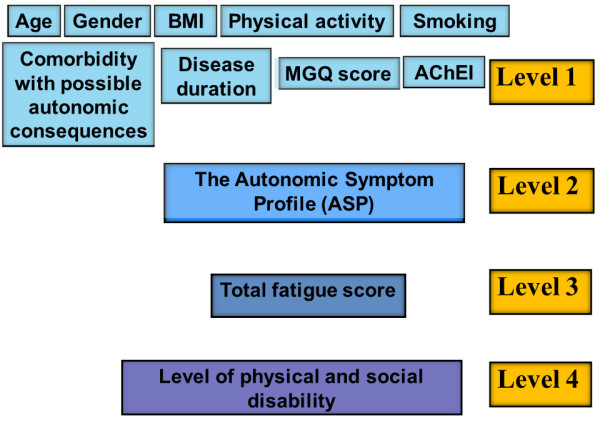
**Estimated analytical model.** We assumed that every variable on a “lower” level could be explained by variables higher in the hierarchy (i.e., variables at Level 4 could be explained by all variables at Levels 1 – 3). *BMI* = body mass index, *AChEI* = acetylcholine-esterase inhibitor.

We constructed an analytical model including all plausible factors as outlined in Figure 1. Based on theoretical plausibility we assumed that variables could be grouped into four “explanatory levels” and that each variable on a lower level could be explained by variables on a higher level in the hierarchy (e.g., variables at Level 4 could be explained by all variables at Levels 1–3).

Variables contained within Level 1 were: age, sex, BMI, physical activity during leisure, smoking, comorbidity, disease duration, MGQ score, use of AChEI, and symptoms related to chronic fatigue syndrome. Level 2 contained the score of the ASP items, Level 3 contained the total fatigue score, and Level 4 contained the functional (physical and social) disability.

Data analysis was performed with SPSS 17.0 software (SPSS Inc., Chicago, IL, USA). The Student *t*-test or the Mann–Whitney *U* test were used as appropriate to compare the MG and control groups, and to compare fatigue level between patients indicated to be symptomatic or asymptomatic according to autonomic symptoms. Variables that strongly deviated from normality were in-transformed to obtain an approximate normal distribution. The potential relationships between the variables within each explanatory level were first explored using bivariate linear regression analyses, and variables with p < 0.1 were included in the multivariate linear regression analyses. In each multivariate model, the distribution of residuals was assessed for normality. For the multivariate regression analyses, we report unstandardized regression coefficients (Bs) with 95% confidence intervals (CIs). A p-value < 0.05 was considered statistically significant.

## Results

Eighty two of the 97 eligible MG patients completed the questionnaires, a response rate of 85%. There were 32 men (mean age ± standard deviation (SD), 59 ± 14 years) and 50 women (mean age, 50 ±16 years). The mean age of all patients was 54 ± 16 years and mean disease duration was 14 ±12 years. Fifty one patients had early onset MG and 31 had late onset MG. Two patients had thymoma-related MG and both underwent surgery with a very good outcome. Mean BMI was 26.5 ± 5.7 kg/m^2^. Of the 78 patients with AchR antibody data, 63 (81%) were seropositive for AchR antibodies. Eight of the 15 AchR seronegative patients were tested against MuSK (muscle specific tyrosine kinase), and all were negative. All patients were MGFA grade II or better during the year prior to the start of this study. Fifty patients (61%) used an AChEI (pyridostigmine bromide) with maximum dose 120 mg per day, and 39 patients (48%) used oral steroids. Fifteen patients took azathioprine, two mycophenolate mofetil, one cyclosporine and one methotrexate. Twenty one patients (26%) had no medication for at least 6 months, either because of complete spontaneous remission after thymectomy or because they had minimal symptoms which did not need treatment. The controls included 410 age- and sex-matched ethnic Norwegians, 160 men (mean age, 59 ± 14 years) and 250 women (mean age, 50 ± 16).

### Fatigue prevalence

MG patients had higher total fatigue scores of 14.5 ± 5.5 compared with 12.4 ± 3.6 in controls (p = 0.001). Patients also had higher physical fatigue scores of 9.8 ± 4.2 compared with 8.0 ± 3.0 in controls (p = 0.001). The level of mental fatigue in patients was 4.7 ± 1.9 *versus* 4.5 ± 1.3 in controls (p = 0.03). Fatigue (total, physical and mental) showed no significant difference between males and females in the patient group.

**Figure 2:**
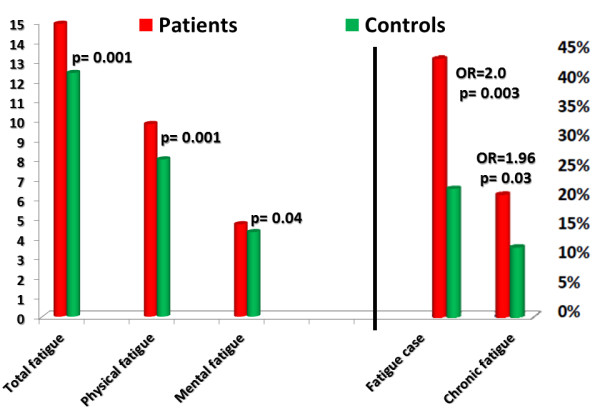
Fatigue score and the prevalence of fatigue caseness and chronic fatigue among myasthenia gravis patients and controls.

Forty-four per cent (36/82) of patients fulfilled the criteria for fatigue caseness (dichotomized score ≥ 4) *versus* 22% (90/410) in controls (odds ratio: 2.0; p = 0.003). Chronic fatigue (dichotomized score ≥ 4 and symptoms lasting 6 months or longer) occurred in 21% (17/82) of patients compared with 11% (48/410) of controls (odds ratio: 1.96; p = 0.03). Figure 2 illustrates the difference in fatigue levels and the prevalence of fatigue caseness and chronic fatigue among MG patients and controls.

### Bivariate and multivariate analysis

Of the explanatory variables in Level 1 (Figure 1), no independent associations with fatigue level were found for patients’ age, gender, BMI, disease duration, physical activity or smoking. On the other hand, the MGQ score and comorbidity showed a correlation with fatigue level.

**Figure 3:**
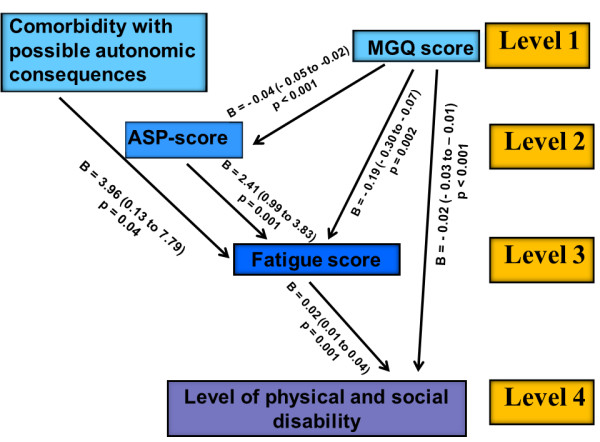
**Results of multivariate analysis.** The results are presented as regression coefficient B (95% confidence interval). *ASP* = autonomic symptoms profile.

A significant negative linear correlation was found between the MGQ score and the ASP score, indicating more autonomic symptoms among the patients most severely affected by MG (Figure 3). The total fatigue score was independently associated with comorbidity and the scores for MG severity and ASP. The functional level was associated with both the MG score and the total fatigue score.

### Prevalence of fatigue in relation to each autonomic symptom

We stratified the patients into symptomatic and asymptomatic groups according to each autonomic symptom. We compared the prevalence of fatigue caseness and the total fatigue score between symptomatic and asymptomatic groups for each autonomic symptom. Symptomatic MG patients had significantly higher fatigue score and prevalence of fatigue caseness for each autonomic symptom, apart from gastrointestinal dysfunction. The difference was especially highly significant for symptoms of sleep disturbance and sudomotor/thermoregulation dysfunction (p = 0.001) as shown in Table 1.

**Table 1:** Distribution of fatigue caseness and total fatigue levels among myasthenia gravis patients by type of autonomic symptom and presence or absence of that symptom

**Autonomic symptom**	**All patients** **(n = 82)**	**Fatigue cases** **(n = 36)**	Odds ratio (95% CI)*	**Total fatigue level**	**p-values**
Sudomotor	Symptomatic	26		17.0	
	(n = 41)		5.4 (2.1 – 14.0)		0.001**
	Asymptomatic	10		12.0	
	(n = 41)				
Sleep	Symptomatic	22		16.8	
disturbances	(n = 38)		2.9 (1.2 – 7.3)		0.001**
	Asymptomatic	14		12.5	
	(n = 44)				
Orthostatism	Symptomatic	20		16.1	
	(n = 36)		2.3 (1.0 – 5.7)		0.04**
	Asymptomatic	16		13.3	
	(n = 46)				
Vasomotor	Symptomatic	15		16.5	
	(n = 26)		2.3 (0.9 – 5.9)		0.03**
	Asymptomatic	21		13.6	
	(n = 56)				
Gastrointestinal	Symptomatic	9		16.3	
	(n = 18)		1.4 (0.5 – 4.0)		ns
	Asymptomatic	27		14.0	
	(n = 64)				

* 95% Confidence interval for Odds ratio.

** The p-values were not adjusted for multiple testing.

## Discussion

The results confirmed our hypothesis that there is an association between MG severity and symptoms of autonomic disturbance, and these in turn are associated with fatigue level and functional disability. Moreover, the most important finding is that the fatigue levels in MG patients, independent of their MG score, were significantly associated with the scores of the autonomic symptoms (p = 0.001). The strongest relationships were found between fatigue and poor thermoregulation, orthostatism and poor sleep. As expected, the frequency and the level of fatigue was significantly higher in MG patients compared with controls. A previous study of 28 MG patients reported subjective fatigue in 82%, and half of these patients experienced cognitive as well as physical fatigue [7]. Our study showed a prevalence of fatigue caseness of 44%. However, our patients did not have severe MG (MGFA 0-II) and elderly patients > 74 years were excluded to make the study comparable with normative data. Furthermore, about 50% more MG patients (21% *vs* 12%) reported chronic fatigue than controls. The main strength of the present study is the relatively large sample of both patients and controls, and the standardized measure of fatigue caseness and chronic fatigue.

The causes of fatigue in MG are difficult to interpret in clinical practice. Myasthenic muscle fatigability is a central factor in MG diagnosis. A previous study showed inconsistency between the subjective feeling of fatigue and the MG-associated muscle fatigability proven by neurophysiological tests such as repetitive nerve stimulation [8]. Disease severity is the most important contributory factor to fatigue in MG patients. However, we found that even MG patients in complete stable remission without detectable myasthenic weakness or medication also reported significantly higher levels of fatigue than controls suggesting that factors other than myasthenic muscle weakness may be involved.

Our study found no clear gender difference in the frequency and level of fatigue in MG patients. In the general Norwegian population, women had higher fatigue levels than men [16]. The dominance of females among MG patients could suggest that gender might be a contributing factor to the high fatigue score, but the bivariate analysis did not support this theory. One study reported that MG patients had a higher BMI due to steroid use, which could reduce their physical activity and potentially be an explanation for fatigue [3]. In that study, the mean ± SD patient BMI was much higher than in our study 31.8 ± 6.8 *vs* 26.5 ± 5.7 kg/m^2^. We did not find associations between BMI or physical restriction and fatigue or autonomic symptoms. Medication can influence both fatigue and autonomic functions. We concluded in a previous study that well-regulated MG patients with fatigue showed an obstructive pattern in pulmonary function tests and this was more pronounced in patients using AChEI [23]. Sixty one per cent (50/82) of the MG patients in our study used AChEI, which is suspected to alter autonomic functions, but we did not find that use of AChEI was an independent explanatory variable, although patients using them had slightly higher total fatigue scores than controls (mean ± SD: 15.0 ± 6.2 *vs* 13.8 ± 4.2). The influence of corticosteroids or immunosuppressives did not reach statistical significance in the bivariate analysis.

The difference in fatigue level was highly significant between patients with symptoms related to thermoregulatory and sleep disturbances compared with those without (Table 1). The frequency of sleep problems in our patients was 46%. Comparable results were found in a newly published study [24], which reported significant sleep disturbance in 59% of 54 clinically stable MG patients. Others reported reduced REM sleep in MG patients with sleep disorders [25,26] and impaired memory in MG patients with sleep apnoea [27].

The correlation between fatigue and ASP scores support previous reports which indicated an autonomic disturbance in patients complaining of fatigue [9,10]. Poor sleep quality and orthostatic intolerance have both been reported in MG patients [11,28]. However, these findings were not discussed in the context of fatigue in these patients. It is reasonable to address sleep disorders in MG as it might be expected that they could aggravate fatigue.

There are some limitations in the present study. There was no adjustment for depression, as there is an overlap in symptomatology between depression and fatigue. The cross-sectional design precluded a possible causal relationship among variables. The impairment in muscle strength in MG necessitates mental concentration for daily activities which are normally done automatically. Therefore, it is difficult to assess purely mental fatigue in MG patients. An important limitation of this study is that the ASP questionnaire has not been formally validated in the MG population. However, this questionnaire provides a screening tool for patients with MG, and objective physiological tests should be used in further studies. Some of the symptoms assessed in this score may also be interrelated with neuromuscular manifestations, such as pupillary reactivity in the ASP that may overlap with diplopia in the MGQ. Our findings show an association between autonomic symptoms and fatigue, but are not proof of causality. We applied the characteristics of fatigue in CFS as a working definition for this study; however, it remains an open question whether the mechanism behind the fatigue is identical in these two separate conditions. Our results can be used as a framework for further analysis. The use of a self-report questionnaire may have contributed in some degree to the extent of the relationships that were observed.

Fatigue has been under-recognized in MG patients. In the absence of a biomarker which could give an objective evaluation of disease severity, patient assessment was based on the patient’s perspective of their symptoms and a clinical examination. Autonomic symptoms, especially thermoregulatory and sleep disorders and fatigue should be taken more seriously than they usually are in these patients. Fatigue could be misinterpreted as myasthenic weakness and trigger a negative spiral, i.e., overmedication with AChEI, which in turn could aggravate fatigue.

## Conclusion

Our results indicate that fatigue is a considerable problem in MG even in patients who are in full remission and not taking medication. MG severity is highly suggestive to be associated with symptoms of autonomic disturbance, which in turn are associated with total fatigue level and functional disability. Future research is needed to understand the role and the mechanisms of autonomic dysregulation in MG-related fatigue. New management strategies should be explored to improve fatigue in MG patients.

## Competing interests

The authors declare that they have no competing interests.

## Authors’ contributions

AE:- Substantial contribution to conception and study design. Collecting of data. Clinical assessment of MG patients. Analysis and interpretation of data. Drafting the article. Final approval of the version to be published. VBW:- Participated in study design. Participated in data analysis. Final approval of the version to be published. JHL:- Provided the epidemiological data fatigue in controls. Participated in data analysis. Final approval of the version to be published. EK:- Substantial contribution to conception and study design. Clinical assessment of MG patients. Revising the article critically. Final approval of the version to be published.

## Pre-publication history

The pre-publication history for this paper can be accessed here:

http://www.biomedcentral.com/1471-2377/13/132/prepub
